# Meis2 is essential for cranial and cardiac neural crest development

**DOI:** 10.1186/s12861-015-0093-6

**Published:** 2015-11-06

**Authors:** Ondrej Machon, Jan Masek, Olga Machonova, Stefan Krauss, Zbynek Kozmik

**Affiliations:** Institute of Molecular Genetics, The Czech Academy of Sciences, 14200 Praha, Czech Republic; Unit for Cell Signaling, Oslo University Hospital, N-0349 Oslo, Norway

**Keywords:** Meis2, Neural crest, Persistent truncus arteriosus, Craniofacial skeleton, Cranial nerves

## Abstract

**Background:**

TALE-class homeodomain transcription factors Meis and Pbx play important roles in formation of the embryonic brain, eye, heart, cartilage or hematopoiesis. Loss-of-function studies of Pbx1, 2 and 3 and Meis1 documented specific functions in embryogenesis, however, functional studies of Meis2 in mouse are still missing. We have generated a conditional allele of Meis2 in mice and shown that systemic inactivation of the Meis2 gene results in lethality by the embryonic day 14 that is accompanied with hemorrhaging.

**Results:**

We show that neural crest cells express Meis2 and Meis2-defficient embryos display defects in tissues that are derived from the neural crest, such as an abnormal heart outflow tract with the persistent truncus arteriosus and abnormal cranial nerves. The importance of Meis2 for neural crest cells is further confirmed by means of conditional inactivation of Meis2 using crest-specific AP2α-IRES-Cre mouse. Conditional mutants display perturbed development of the craniofacial skeleton with severe anomalies in cranial bones and cartilages, heart and cranial nerve abnormalities.

**Conclusions:**

Meis2-null mice are embryonic lethal. Our results reveal a critical role of Meis2 during cranial and cardiac neural crest cells development in mouse.

**Electronic supplementary material:**

The online version of this article (doi:10.1186/s12861-015-0093-6) contains supplementary material, which is available to authorized users.

## Background

Neural crest cells (NCC) represent a multi-potent embryonic cell population that generates a very diverse range of cell types including cranial nerves, neurons and glia of the peripheral nervous system, enteric neurons, melanocytes, cranial bones and cartilages [1, 2]. The first NCC appear at the neurula stage in the neural plate border region. As the neural tube closes in mouse, NCC delaminate from the regions of neural plate border and ectomesenchyme after epithelial-to-mesenchymal transition (EMT) and migrate to various developing organs. The very broad differentiation potential of NCC provides a complex model of cell type specification and migration and the gene regulatory network determining the spatiotemporal control of NCC diversification has been extensively studied. For instance, the NCC population is specified by the set of transcription factors Sox9, Sox10, FoxD3, Snai2 together with Msx1, Pax3/7 or Zic1 in the neural plate border [3]. These effector genes are regulated by coordinated action of signaling pathways such as Wnt, Bmp and Fgf from the adjacent paraxial mesoderm and non-neural ectoderm [2, 4]. The differentiation potential of NCC is spatially determined by their position along the rostrocaudal axis. In a simplified view, cranial NCC coming from mesencephalic and rhombencephalic regions generate head bones, cartilages, cranial nerves and selected connective tissues [5, 6]. Vagal NCC from the area of somites 1-7 are destined to the enteric nervous system. Cardiac NCC (somites 1-4) are involved in septation of the cardiac outflow tract [7] and trunk NCC form sensory and sympathetic ganglia. The current debate, however, favors the scenario proposing that originally multi-potent NCC stem cells are exposed to different environmental cues along the rostrocaudal axis that spatiotemporally restrict their differentiation potential [1, 8].

Meis proteins are transcription factors that are orthologous to the *Drosophila* homothorax (Hth) protein. They contain a TALE (three-amino-acid loop extension) sub-class of the homeodomain that binds to DNA. In humans and mice, three homologues Meis1, Meis2 and Meis3 have been identified [9] and it has been shown that they directly bind to Pbx proteins [10–12]. The Meis/Pbx protein complex binds to DNA through respective Meis- and Pbx-consensus binding sites thereby regulating transcription. The Meis/Pbx complex plays important roles during development of several organs including limbs [13, 14], heart [15, 16], lens [17], pancreas [18] and hindbrain [19–22]. Hox genes are among the target genes of Meis-Pbx control via modulation of histone acetylation indicating recruitment of Hox proteins as cofactors of Meis-Pbx complex [23, 24].

Mice lacking Meis1 display liver hypoplasia, hemorrhage, impaired erythropoiesis and eye defects, and die by the embryonic day (E) 14.5 [25, 26]. Although a substantial amount of data have been reported on the role of Meis1 in organogenesis, hematopoiesis and leukemia induction, the function of the other homologs, Meis2 and Meis3, is much less clear. Chicken Meis2 has a specific role in determining cell fate in the midbrain-hindbrain boundary by controlling the expression of Otx2 [22] and it also affects proliferation of retinal progenitor cells [27]. Several recent reports in various model systems indicated that Meis2 may play a role in neural crest cells. Meis2 was identified as one of the key transcription factors in the gene regulatory network driving differentiation of human embryonic stem cells towards cardiovascular cell types, and this was further confirmed by knock-down experiments in zebrafish [16]. Morpholino-based screens in zebrafish revealed the importance of Meis1 and Meis2 factors during craniofacial development [28]. Moreover, gene expression analysis of EMT in endocardial cushions identified Meis2 among enriched genes [29]. In this context it is very interesting that some human disorders displaying cleft palate and heart developmental defects have been linked to mutations in the Meis2 locus [30–33]. Nonetheless, a clear picture of the Meis2 function based on a genetic mouse model is still missing.

In the present study, we examined the role of Meis2 during embryogenesis by generating conditional knock-out mice. We studied morphological defects after either zygotic inactivation of the Meis2 allele or NCC-specific conditional knock-out using AP2α-IRES-Cre. We conclude that hemorrhaging most probably causes embryonic lethality. Further, many embryonic defects in the tissues derived from neural crest in systemic Meis2-nulls were recapitulated upon conditional deletion of Meis2 in NCC suggesting an indispensable role of Meis2 in NCC.

## Results

### Meis2-/- embryos are lethal and display hemorrhaging

**Fig. 1:**
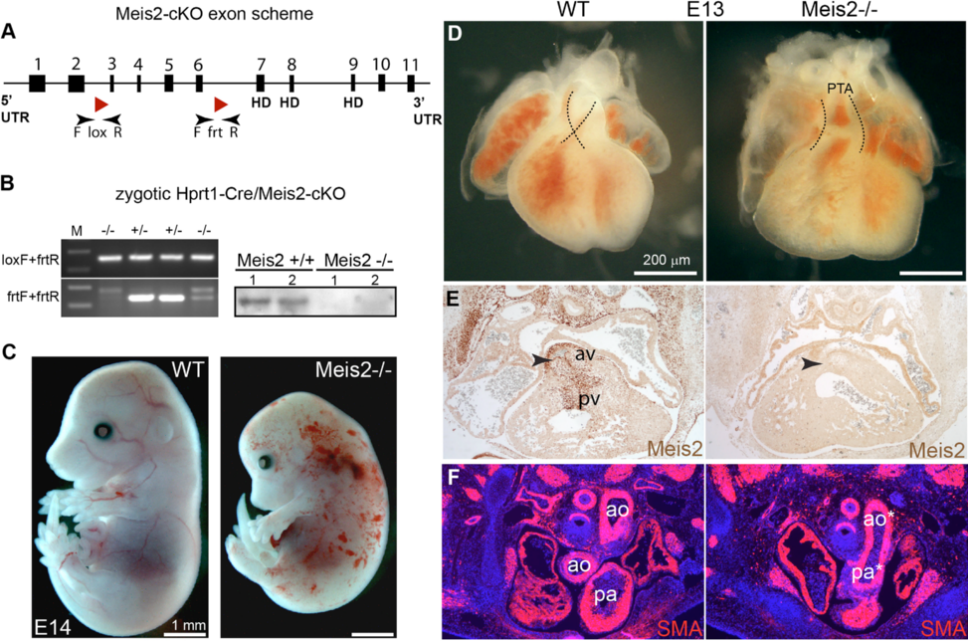
Generation of Meis2 -/- mice. **a** Scheme of the *Meis2* gene with 11 exons (black rectangles) and position of loxP sites for Cre recombination. The homeodomain (HD) is encoded by exons 7-9. **b** Meis2-null (-/-) embryos were obtained by crossing Meis2 cKO with Hprt1-Cre mice. Left: Genotyping of Meis2-/- embryos using primers loxF, frtF and frtR illustrated in (**a**) (see also Methods). Right: Western blot analysis of E12.5 whole-embryo protein extracts with an anti-Meis2 antibody. **c** Mutant Meis2 mouse embryos at E14.5 display severe hemorrhaging. **d** The embryonic heart at E13 with persistent truncus arteriosus (PTA) in which the aortic and pulmonary arteries are not separated in Meis2-/-. A dashed line depicts the crossing of the separated aorta and pulmonary artery in the wild-type heart while this is not visible in the mutant outflow tract. **e** Immunohistochemical staining of Meis2 in the embryonic heart at E13 (transverse sections) illustrating its strong expression in the aortic (av) and pulmonary valves (pv). Aortic and pulmonary valves are missing in Meis2 mutants (arrowhead). **f** α-Smooth muscle actin (SMA) staining on transverse sections showing persistent truncus arteriosus with the fused aorta (ao*) and pulmonary artery (pa*) in Meis2-/-. Ao, ascending and descending aorta; pa, pulmonary artery; PTA, persistent truncus arteriosus

A conditional mutant allele of the *Meis2* gene (Meis2 cKO) was created by inserting LoxP sites in the introns 2 and 6 that flank exons 3 and 6 in the *Meis2* gene (Fig. 1a). To generate mutant mice lacking functional Meis2 in the whole organism, Meis2 cKO were at first crossed with the Hprt1-Cre mice (Jax Mice and Services) that exert a zygotic expression of *Cre* recombinase facilitating gene excision in all tissues. The first generation mice, which were heterozygous for the Meis2 gene (Meis2+/-), were intercrossed to obtain Meis2-null (Meis2-/-) animals. Animals were genotyped using primers flanking loxP sites as depicted in Fig. 1a. The loss of exons 3-6 in Meis2-/- embryos was tested by PCR (Fig. 1a, left). The absence of Meis2 protein was verified using Western blot analysis of protein extracts from E12.5 embryos (Fig. 1b, right). Meis2-/- mice displayed embryonic lethality between E13.5-E14.5 and suffered from hemorrhaging (Fig. 1c). The size of mutant embryos was smaller at E14.5 as mutant embryos stopped growing approximately at E13.5 when hemorrhaging became prominent. Although the severity of this phenotype varied among Meis2-/- (n = 29 litters, 51 mutants), all mutants displayed bleeding and a small liver size (Additional file 1: Figure S1B).

A detailed inspection of internal organs in mutant embryos revealed that the liver was the most impaired organ with a destructed cellular organization in large regions (Additional file 1: Figure S1C-D). These impaired regions contained almost no erythrocytes labelled with Ter119 but many apoptotic cells as shown by TUNEL assay (Additional file 1: Figure S1E-F). Surprisingly, Meis2 was not found to be expressed in the fetal liver while Meis1 was readily detectable (Additional file 1: Figure S1A). Based on this we suggest that the observed cell death in the liver is a consequence of strong hemorrhaging in the whole embryo that leads to anemia and apoptosis primarily in the liver and may be a cause of the embryonic lethality. Having observed anemia in Meis2-/- embryos we further pursued the possibility that Meis2 may influence embryonic hematopoiesis similarly to Meis1 that controls proliferation of hematopoietic stem cells in the fetal liver and is also essential for megakaryocyte viability [25, 26, 34]. We therefore mapped the expression of Meis2 and Meis1 in the area of the aorta-gonad-mesonephros (AGM), the site of origin of embryonic hematopoietic stem cells. As shown in Additional file 1: Figure S2B, neither Meis2 nor Meis1 were observed in endothelial cells labelled with CD31 but both proteins were abundant in the mesenchyme surrounding the endothelial wall of the dorsal aorta. We further found that circulating hematopoietic progenitors labeled with anti-Runx1 antibody did not express Meis2 (Additional file 1: Figure S2C). Finally, we carried out erythroblast cultures derived from the fetal liver and found no differences in the growth and differentiation of liver erythroid progenitors between Meis2-/- and controls (data not shown). As Meis2, in our hands, was not detected in hematopoietic progenitors neither in the AGM nor in the fetal liver, we hypothesize that the anemia in the mutants originates from extensive bleeding or defective circulation rather than defects in hematopoiesis.

### The lack of Meis2 results in fetal heart malformation

In the heart of Meis2-/- embryos at E12.5, we observed incomplete septation of the outflow tract that normally separates the aorta from the pulmonary artery. This defect is known as persistent truncus arteriosus (PTA) and was observed in all analyzed mutants (n = 8 litters, 14 mutants) (Fig. 1d). To correlate the observed defects with the expression of Meis2, we carried out immunohistochemistry on sections of embryonic heart at E13 using anti-Meis2 antibody. As shown in Fig. 1e, a remarkably strong presence of Meis2 was observed in the aortic and pulmonary valves. Strikingly, these valves were lost in the Meis2-/- heart (arrowheads). To visualize the PTA in the Meis2-/- outflow tract, we used antibody against α-smooth muscle actin (SMA). Stained heart sections confirmed the PTA but SMA appeared normally expressed in the mutant heart (Fig. 1f).

**Fig. 2:**
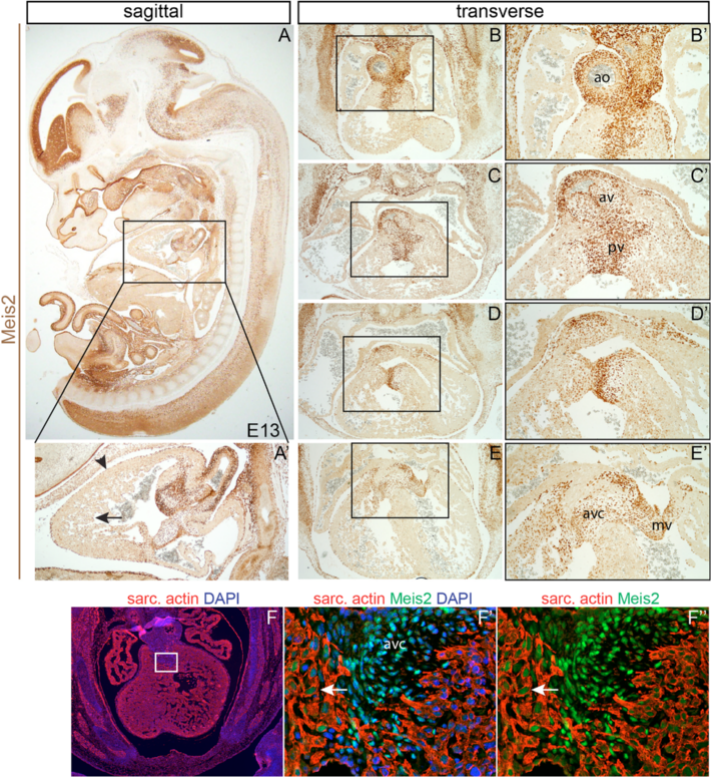
Mapping of Meis2 expression in the mouse embryo at E13. Meis2 protein is present in many developing tissues including the forebrain, midbrain, hindbrain, spinal cord, myocardium, cardiac cushions, cardiac outflow tract, valves (aortic, pulmonary, tricuspid, mitral). (**a**) A sagittal section of the whole embryo with a detail of the heart (A’) documenting Meis2 presence in the myocardium (arrow) and pericardium (arrowhead). (**b-e**) Transverse sections of the heart at E13 from rostral to caudal with details of the framed areas (**b’-e’**) illustrating strong Meis2 expression in cardiac valves. (**f**) Immunofluorescent labeling of sarcomeric actin in the wild-type heart at E13. (**f’-f”**) Double labeling of sarcomeric actin (red) and Meis2 (green), a higher magnification view on the framed area in (**f**) showing Meis2 in the myocardium (arrows) and in the atrioventricular cushion. ao, aorta; av, aortic valve;, pv, pulmonary valve; mv, mitral valve; avc, atrioventricular cushion

Widespread expression of Meis2 was observed in the developing embryo including the central nervous system, in the upper and lower jaw and in the lumen of the intestinal tract (Fig. 2a). Specifically in the heart, Meis2 protein was detected in the myocardium (arrow) and pericardium (arrowhead) (Fig. 2a’) in which Meis2-positive cells also expressed sarcomeric actin (Fig. 2f-f”, arrows). Remarkably strong expression of Meis2 was found in the valves (aortic, pulmonary, tricuspid and mitral) as well as in the atrioventricular cushion (Fig. 2b-f”).

**Fig. 3:**
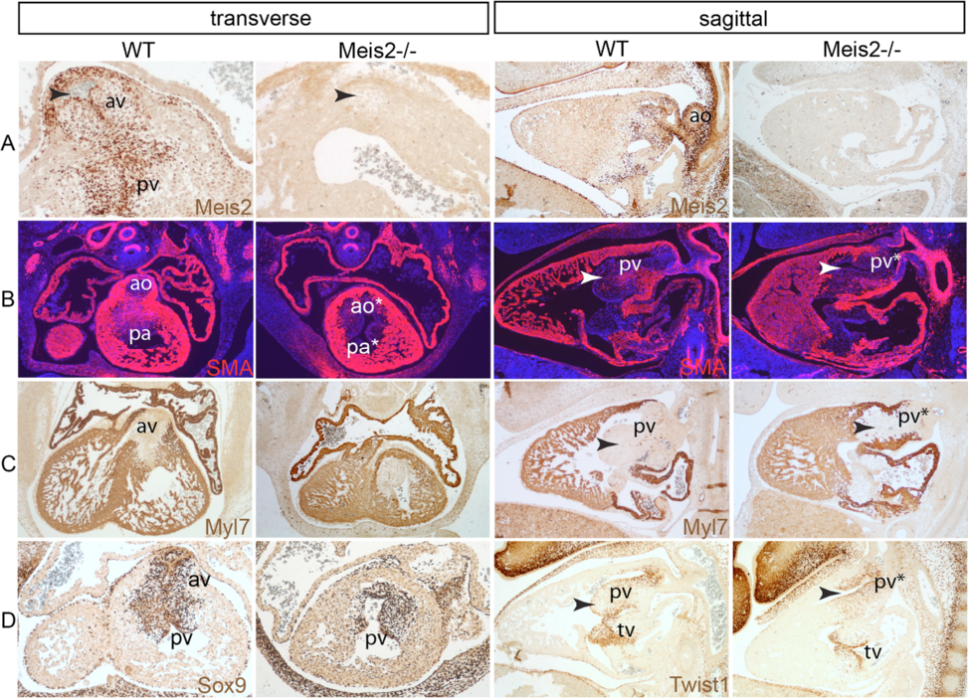
Immunohistochemistry of transverse and sagittal sections at E13.5 from the embryonic heart in Meis2-/- mice and controls. **a** Anti-Meis2 antibody staining (brown) show strong expression in the pulmonary and aortic valve. The aortic valve is absent in the mutants lacking Meis2 (arrowhead). **b** α-Smooth muscle actin (SMA) staining showing the persistent truncus arteriosus (PTA) with the fused aorta and pulmonary artery (presumptive ao* and pa*). Sagittal sections document a shortened heart length and a malformed pulmonary valve. **c** Heart-specific myosin light chain 7 (Myl7) staining shows normal cell specification in the myocardium in Meis2 nulls and, again, absent aortic and abnormal pulmonary valve on transverse and sagittal sections, respectively. **d** Left: Decrease of Sox9-positive cells in the valves in the outflow tract in Meis2 nulls. Right: The level of Twist1 expression in the condensed mesenchyme in the pulmonary valve and in the tricuspid valve appears normal in the mutants but it clearly reveals severe morphological changes in OFT. av, aortic valve; pv, pulmonary valve; tv, tricuspid valve; ao, aorta; pa, pulmonary artery

To document heart defects in Meis2-/- embryos in detail, we performed immunofluorescence on transverse and sagittal sections from embryos at E12.5 using antibodies against Meis2, α-smooth muscle actin (SMA) and heart-specific myosin Myl7. Figure 3a-c illustrates that the aortic and pulmonary arteries were not separated (ao*, pa*, pv* in Fig. 3b) and the aortic valve was absent (arrowhead in Fig. 3a). These regions strongly expressed Meis2 in the controls and the signal was lost in the mutants (Fig. 3a). Both the aortico-pulmonary spiral septum and the aortic and pulmonary valves are formed from from cardiac neural crest cells [7] which can be visualized by anti-Sox9 and anti-Twist1 antibody [35]. In Meis2-/- embryonic hearts, we observed less Sox9-positive cells in the outflow valves. The number of Twist1-positive cells was not significantly changed though its expression level was reduced and the organization of these cells was altered compared to controls (Fig. 3d). Clear morphological defects in the septation of the truncus arteriosus and in the outflow valves (arrowheads) strongly suggest that the defective outflow tract in Meis2 nulls arises from impaired cardiac neural crest cells.

### Neural crest cells express high levels of Meis2

**Fig. 4:**
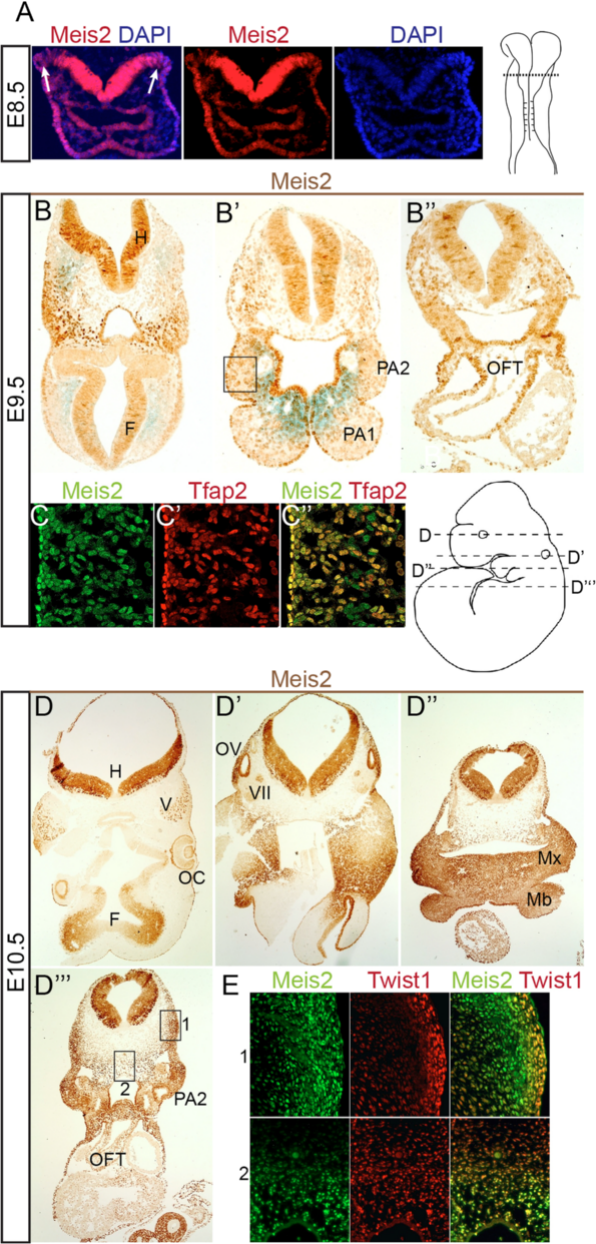
Meis2 is expressed in migrating NCC. (**a**) Meis2 expression in the neural plate stage at E8.5 becomes weak towards the neural plate border (arrows). Right: schematic embryo at E8.5 with a depicted section plane (dashed). (**b-b”**) Sections from rostral (**b‘**) to caudal (**b‘’**) of wild-type embryos at E9.5 revealing Meis2 in pharyngeal arches (PA), hindbrain (H) and in the cardiac outflow tract (OFT). (**c-c”**) Immunofluorescent double-labeling of Meis2 (green) and Tfap2 (red) in the region approximately depicted by a black rectangle in A’. (**d-d”’**) Transverse sections from rostral to caudal at E10.5 with immunostained Meis2. Sections levels correspond to the illustrated cartoon. Meis2 was detected in the hindbrain (H), forebrain (F), optic cup (OC), trigeminal nerve ganglion (V), otic vesicle (OV), facial nerve ganglion (VII), maxillary (Mx) and mandibular (Mb) process of PA1, in PA2 and outflow tract (OFT). (**e**) Immunofluorescence of Meis2 (green) and Twist1 (red) shows their co-expression in two areas labelled with rectangles in D”’. Upper panels (1) belong to the lateral zone (1) and lower panels (2) to the midline area (2)

Based on the heart abnormalities of potential neural crest origin, we decided to map the expression of Meis2 at the embryonic stages that are critical for generation and migration of NCC. At E8.5 before the neural tube closure, abundant Meis2 expression was noticed in the neural plate that weakened towards the plate border from which the premigratory NCC delaminate (Fig. 4a). At E9.5, the Meis2 protein was found in the neuroepithelium of the hindbrain (Fig. 4b-b”) in which the signal became diminished towards the roof plate. Meis2 was also seen in migrating cells in the mesenchyme lateral to the hindbrain and in the pharyngeal arches 1-2 (PA1 and PA2) (Fig. 4b’) and in the outflow tract (OFT) (Fig. 4B”). Many of these cells especially in the PA co-express Tfap2, a marker of NCC [36, 37] (Fig. 4c-c” and Additional file 1: Figure S3C-D”). At E10.5, Meis2 protein was detected in the developing tissues that are derived from NCC, such as the trigeminal and facial nerve ganglions, in the the otic vesicle and its surrounding mesenchyme, in the maxillary and mandibular component of the PA1, in the cardiac outflow tract cushion and in the PA2 (Fig. 4d-d”’ and Additional file 1: Figure S3A-B”). The expression pattern revealed by our immunohistochemistry thus corresponds to in situ hybridization of Meis2 mRNA reported by Cecconi and co-workers [38]. Apart from the neuroectoderm, many of Meis2-positive cells displayed a mesenchymal character as revealed by co-staining with the anti-Twist1 antibody (Fig. 2E and Additional file 1: Figure S3E).

### Conditional deletion of Meis2 in neural crest cells leads to a defective heart outflow tract

**Fig. 5:**
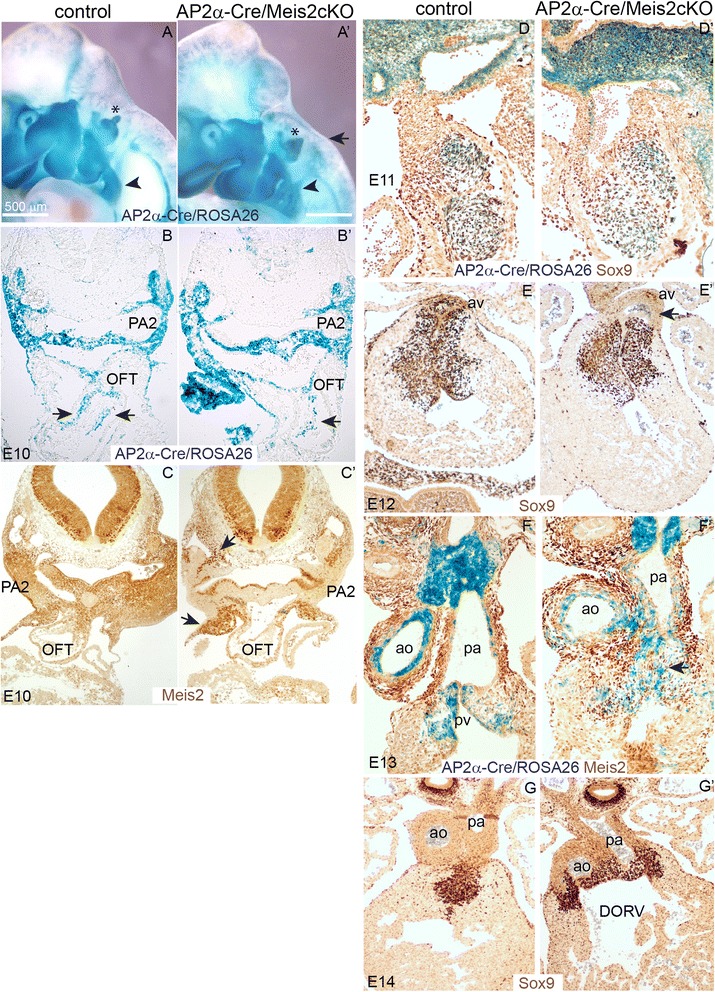
Heart defects upon conditional inactivation of Meis2 in NCC. (**a-a’**) Lineage tracing of NCC in AP2α-Cre /Meis2 cKO/ROSA26 embryos at E10.5 (**a’**) and controls (**a**). (**b-b’**) Sections of embryos shown in (**a**) at the level of PA2 and OFT, compare position and number of β-galactosidase-positive cells in the control and the mutant cKO (arrows in B’). (**c-c’**) Immunohistochemical staining of Meis2 in cKO (**c’**) shows the areas of its deletion. (**d-d’**) Cryosetions of the OFT from AP2α-Cre /Meis2 cKO/ROSA26 stained for β-galactosidase and Sox9, note cells disorganization in cKO (**d’**). (**e-e’**) Paraffin-embedded sections of E12 heart stained with anti-Sox9 defective aortic valve in cKO (**e’**). (**f-f’**) Meis2 and β-galactosidase double-labeling of E13 OFT shows a number of Meis2-positive cells in cKO (**f’**) that are excluded from Cre recombined areas. The pulmonary valve does not develop properly. (**g-g’**) anti-Sox9 immunohistochemical labeling of severed valves in cKO at E14 (**g’**). Ao, aorta; av, aortic valve; OFT, outflow tract; PA2, pharyngeal arch 2; pa, pulmonary artery, DORV, double outlet right ventricle

Because some developmental defects in Meis2 nulls may arise from affected NCC, we aimed to verify this assumption by means of conditional inactivation of Meis2. We employed AP2α-IRES-Cre (AP2α-Cre) transgenic mouse in which Cre recombinase was inserted into the Tfap2 (AP2α) gene enabling loxP recombination specifically in NCC [39]. Conditional mutants AP2α-Cre/Meis2 cKO survived the critical stage E14, at which systemic Meis2 nulls die, but they were lethal around the time of birth. This cKO approach also allowed us to analyze older embryos. To perform lineage tracing experiments and examine migratory routes of NCC, conditional mutants AP2α-Cre/Meis2 cKO were crossed to ROSA26 reporter mice [40]. This allele allows detection of Cre-mediated recombination using β-galactosidase enzymatic assay and tracing of all cells derived from AP2α-Cre positive NCC. Figure 5a-a’ illustrates lineage tracing of NCC in normal and AP2α-Cre/Meis2 cKO embryos. 75 % of mutants (n = 8 litters, 8 mutants) displayed aberrant distribution of β-galactosidase-positive cells along the neural tube (arrow in 5A’), and all mutants had abnormalities in the PA2, which appeared thinner and misshaped, (arrowheads in 5A-A’) and also all mutants had smaller otic vesicles (asterisks). Sectioning of AP2α-Cre/Meis2 cKO/ROSA26 E10.5 embryos revealed poor colonization of the OFT by cardiac NCC (arrows in 5B-B’). Conditional deletion of Meis2 effectively erased the Meis2 protein in the regions of the AP2α-Cre activity, however, many surrounding cells still express normal levels of Meis2 (arrows in Fig. 5c-c’). Next we studied the OFT defects at later stages using immunohistochemistry on cryosections from AP2α-Cre/Meis2 cKO/ROSA26 embryos. At E11, anti-Sox9 antibody labelled all β-galactosidase-positive cells in the OFT and we observed a lower density and disorganization of double-labelled cells in conditional mutants (Fig. 5d-d’). 90 % of mutants had malformed OFT valves at E12 (n = 5 litters, 9 mutants) (such as av in Fig. 5e-e’). However, the septation of the truncus arteriosus appeared normal in conditional mutants as the aorta and pulmonary arteries were normally separated (Fig. 5 f-f’), which was never seen in systemic nulls all displaying the PTA. Of note, we detected still high levels of Meis2 in non-recombined cells, including the smooth muscle of artery walls and in the vicinity of valves, which may provide explanation for less severe defects in conditional mutants compared to systemic ones. Finally, Fig. 5g-g’ shows an example of valve malformation at E14 known as double outlet right ventricle (DORV) labelled with anti-Sox9. In summary, tissue-specific deletion of Meis2 using AP2α-Cre resulted in various valve defects in the heart outflow tract documenting its critical role in cardiac NCC.

### Meis2 affects cranial nerve development

**Fig. 6:**
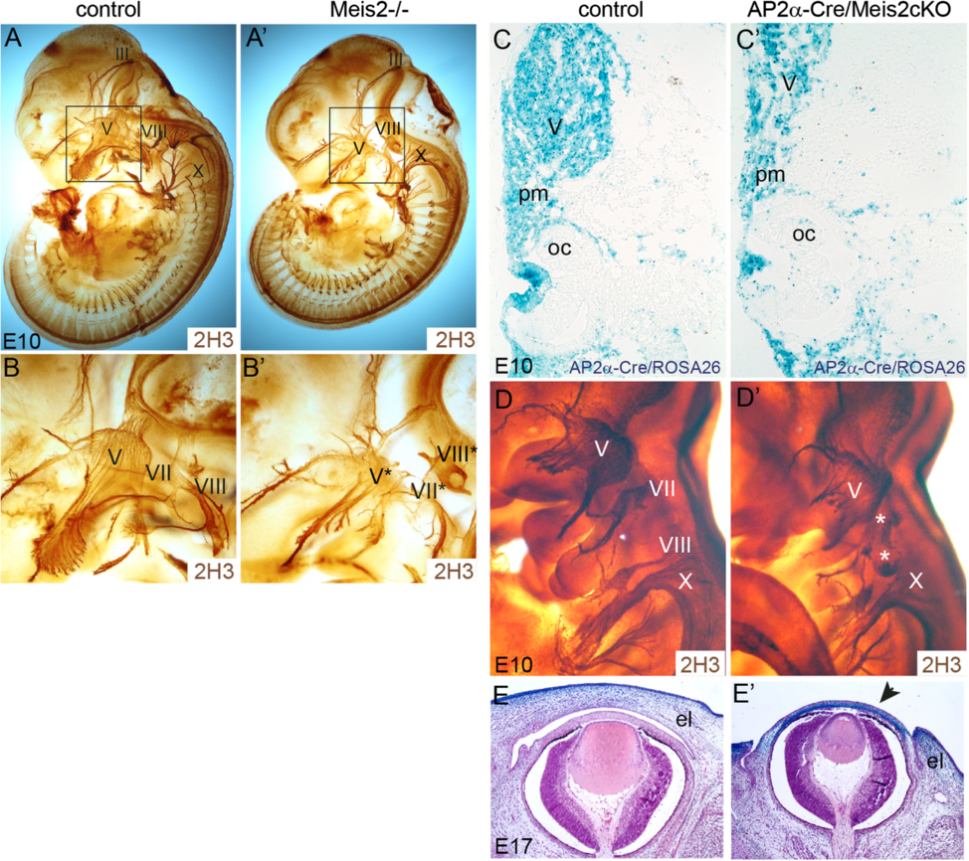
The absence of Meis2 leads to perturbed development of cranial nerves. (**a-a’**) Neurofilament staining using 2H3 antibody revealed absent or abnormally positioned cranial nerves trigeminal V, facial VII and vestibulocochlear VIII in Meis2-/-. (**b-b’**) A detail of the framed areas in (**a**) showing severed nerves V, VII (asterisks). (**c-c’**) Lineage tracing of NCC in AP2α-Cre /Meis2 cKO/ROSA26 embryos at E10.5 and controls. Transverse head sections in the eye area. (**d-d’**) Neurofilament 2H3 staining of cKO embryos at E10.5 with impaired nerves VII and VIII (asterisks). (**e-e’**) Hematoxylin-eosin staining of the eye from cKO at E17 shows shrunken eyelids (el) and cornea (arrow). Cranial nerves III, V, VII, VIII, X; el, eye lid; oc, optic cup; pm, periocular mesenchyme

As Meis2 expression is abundant in the head region, we next explored other NCC derivatives such as cranial nerves. Formation of their neuronal projections in Meis2-/- embryos was followed using whole-mount immunostaining of neurofilaments with the 2H3 antibody. As seen in Fig. 6a-b’, the trigeminal (cranial nerve V), facial (VII) and vestibulocochlear (acoustic) nerves (VIII) were severely impaired in Meis2 nulls at E10.5 (asterisks in Fig. 6b’). An analogous analysis was performed in AP2α-Cre/Meis2 cKO. We again crossed conditional mutants with ROSA26 reporters to map the activity of Cre. β-galactosidase signal in AP2α-Cre/ROSA26 controls was seen in trigeminal ganglions, the lens, the periocular mesenchyme and the surface ectoderm. A similar spatial pattern in this head region was found in the mutants AP2α-Cre/Meis2 cKO/ROSA26 and the size of the trigeminal ganglion V was smaller and misshapen (Fig. 6c’). 2H3 neurofilament staining showed that the cranial nerves VII and VIII were damaged in conditional mutants (asterisks in Fig. 6d’) but the trigeminal nerve V again less affected than in Meis2-/- (n = 3 litters, 3 mutants). Thus, the phenotype in cranial nerves appeared weaker in conditional mutants in comparison with systemic ones. We further noticed that the cornea and eyelids were not developed properly (Fig. 6e-e’) (similarly in Meis2-/- embryos at E13.5, not shown). The cornea was much thinner (arrow) while eye lids (el) did not grow and close over the eye bulbs. The cornea was reported to originate from NCC [41–43] as also indicated by our lineage tracing data showing AP2α-Cre activity in the periocular mesenchyme (Fig. 6c). In summary, tissue-specific deletion of Meis2 in NCC and the surface ectoderm resulted in malformed cranial nerves, the cornea and eyelids.

### Development of cranial cartilage is perturbed in Meis2-null embryos

**Fig. 7:**
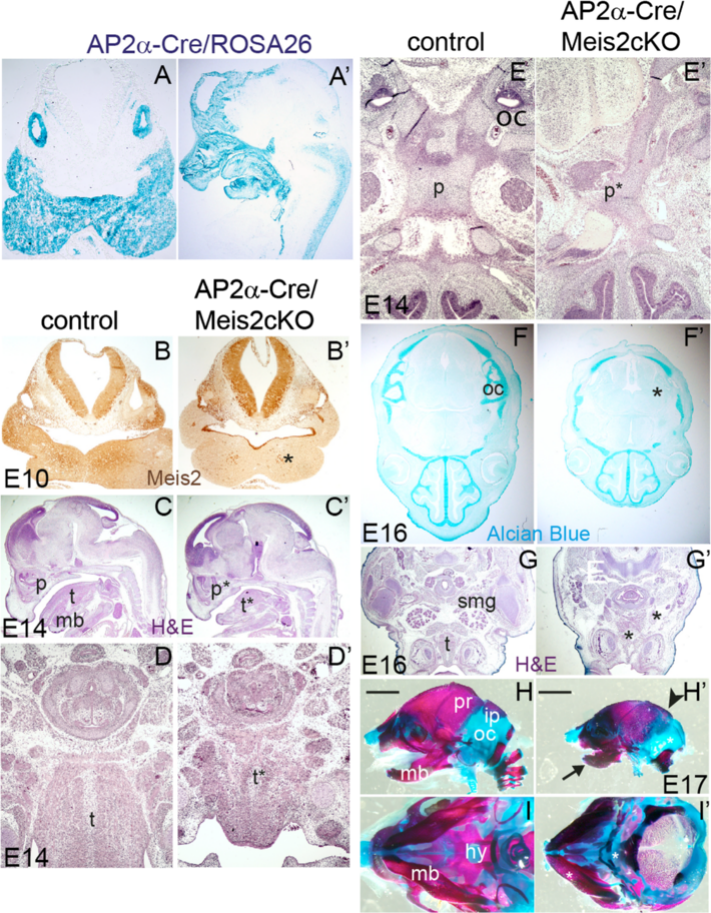
Conditional inactivation of Meis2 in NCC affects osteochondrogenesis in the head. (**a-a’**) Illustration of the zone of Cre activity visualized in AP2α-Cre/ROSA26 embryos, left: transverse section of E10.5 embryo, right: sagittal view on E13.5. (**b-b**) Meis2 immnunostaining of AP2α-Cre/Meis2cKO embryos at E10.5, transverse sections in the region of hindbrain and the first pharyngeal arch. Compare the area of Meis2 deletion with a comparable cryosection from AP2α-Cre /Meis2 cKO/ROSA26 in the left panel (**a**). (**c-c’**) Sagittal sections from cKO at E14, hematoxylin-eosin (H&E) staining. Note the abnormal palate (p*) and tongue (t*). (**d-d’**) A higher magnification of the tongue on transverse sections at E14 documents its much reduced size and tissue disorganization. (**e-e’**) An example of severely disrupted palate (p*) in some cKO embryos, transverse section H&E staining. Alcian staining of cartilage at E16 with no otic capsule in cKO. (**g-g’**) Submandibular gland is almost absent and tongue muscle severed, transverse sections. (**h-h’**) Alizarin Red/Alcian Blue staining of E17.5 heads. Side view with remarkably shorter mandible (arrow), absent interparietal bone, abnormal hyoid bone and otic capsule cartilage. Ossification frontline of the parietal bone is severed in the mutants (arrowhead). (**i-i’**) Ventral views showing an abnormal hyoid bone (asterisk). oc, otic capsule cartilage; ip, interparietal bone; hy, hyoid bone; mb, mandible, p, palate; pr, parietal bone; smg, submandibular gland; t, tongue

Next, we examined the development of the craniofacial skeleton that is derived from NCC. Mapping of AP2α-Cre/ROSA26 showed complete recombination in PA1 and otic vesicle at E10 and this was further verified at E13 on sagittal view in which recombined cells resided in jaws and the nasal cartilage (Fig. 7a). Immunostaining of the Meis2 protein confirmed efficient deletion in AP2α-Cre /Meis2 cKO in the regions of Cre recombination (asterisk in Fig. 7b’, compare with the panel 7a). We found abnormal palate (p) and tongue (t) in sagittal sections of E14 conditional mutants (Fig. 7c-c’). The size of tongue was greatly reduced and muscle fibers looked disorganized, as shown in a detailed view on transverse sections (Fig. 7d-d’). In 33 % of mutants (n = 6 litters, 4 mutants), severely malformed developing cartilage were observed in the palate (asterisk in Fig. 7e’), 67 % of mutants also displayed abnormal palates though less severe. Further, the otic capsule cartilage was absent as shown by Alcian staining at E16 (asterisk in Fig. 7 f’). Submandibular gland (smg) was missing in 33 % mutants and 66 % had much smaller smg (Fig. 7g-g’) (n = 6 litters, 4 and 8 mutants, respectively). We carried out whole-mount Alcian Blue/Alizarin red staining at E17.5 that confirmed our observations from tissue sections: The mandibular bone was poorly developed and its length was much shorter in the mutants (black arrow in Fig. 7h’). The interparietal bone and the cartilage of the otic capsule were absent and the boundary of ossification of the parietal bone was abnormal (arrowhead in Fig. 7h’). The hyoid bone was severely malformed (asterisk in Fig. 7i’). These defects clearly document that Meis2 is essential for the head bones and cartilages that originate from cranial NCC.

### Meis2 does not affect proliferation and expression of main determinants of NCC

**Fig. 8:**
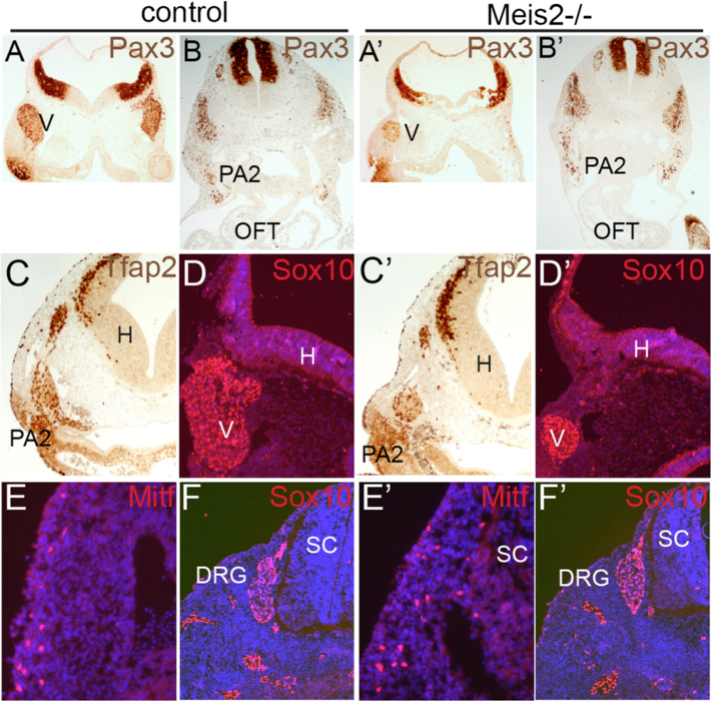
Expression of NCC markers in the absence of Meis2. (**a-a’**) Pax3 immnostaining of trigeminal ganglions in systemic Meis2-/- E10.5 embryos showing greatly reduced or even absent ganglions V. (**b-b’**) Pax3 in the second pharyngeal arch (PA2). (**c-c’**) Tfap2 immunostaining in Meis2-/- embryos at E10.5. (**d-d’**) Sox10 immunofluorescence of ganglions V at E10.5. (**e-e’**) Melanocytes stained with anti-Mitf were detected normally in Meis2 nulls. (**f-f’**) Sox10 in dorsal root ganglia and the peripheral nervous system was not changed in mutants. DRG, dorsal root ganglia; H, hindbrain; V, trigeminal ganglion; PA2, the second pharyngeal arch;, sc, spinal cord

Migrating NCC are exposed to a number of environmental factors that can alter their properties including fate restriction or proliferation. In order to class Meis2 into the gene regulatory network determining NCC development we first examined the levels of expression of well-known genes specifying NCC. Trigeminal ganglions stained with anti-Pax3 were found reduced in size or completely missing in Meis2-/- embryos at E10 (Fig. 8a-A’). Conditional mutants showed normal levels of Pax3 and a normal size of ganglions V (not shown). Pax3 was reported to control colonization of the OFT by cardiac NCC [44] and the promoter of Pax3 is directly controlled by Pbx1 [45]. As Pbx1-/- embryos display similar heart defects as Meis2-/- embryos [15], we checked the expression of Pax3 in the OFT and PA2 of our mutants. However, we found no change in Pax3-positive cells in PA2, moreover, Pax3 was not detected in the OFT even in wild-type controls (Fig. 8b-b’). Next we assayed the level of expression of Tfap2, Sox10 and Mitf, a marker of melanocyte precursors. As shown in Fig. 8c-f’, their expression was not found to be changed in the absence of Meis2.

We also analyzed the number and spatial distribution of migrating NCC using anti-Sox9 antibody at E10.5. Sox9-positive NCC are abundant in the embryonic head mesenchyme and pharyngeal arches, however, their quantification did not reveal significant changes between controls and mutants (Additional file 1: Figure S4A). Further, we examined cell proliferation using antibodies against PCNA and PH3 on head sections. Again, quantification of all proliferating cells (all PCNA+ or PH3+) or proliferating NCC (Sox9+/PCNA+) did not differ between controls and mutants (Additional file 1: Figure S4B-D). Finally, apoptosis, as measured by anti-Cas3 immunohistochemistry at E10.5, was not significantly altered in the mutants (Additional file 1: Figure S4E). In summary, Meis2 absence did not result in dramatic changes in cell proliferation and viability at E10.5.

### Systemic Meis2 mutants show aberrant position of FoxD3 and Sox9

**Fig. 9:**
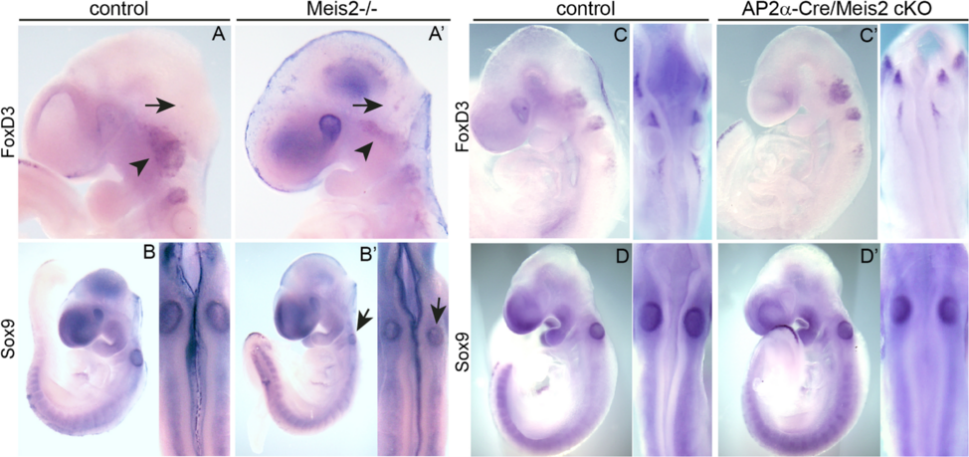
Expression of neural crest cell specifier genes FoxD3 and Sox9 in the absence of Meis2. (**a-a’**) In situ hybridization of FoxD3 on E9.5 Meis2-/- embryos and controls with an abnormal position of migrating NCC in the midline of the midbrain, hindbrain and spinal cord (arrows in side and dorsal views in **a’**). (**b-b’**) *Sox9* mRNA staining at E9.5 with ectopic cells in the midline (arrows in side and dorsal views in (**a’**) and smaller otic vesicles (arrowheads) in systemic mutants. (**c-c’**) In situ hybridization of neural crest cell specifier mRNA *FoxD3* and *Sox9* (**d-d’**) in conditional AP2α-Cre/Meis2cKO at E9.5. Side and corresponding dorsal view magnifications illustrate no change in their expression in conditional mutants

As the defects in NCC derivatives in the absence of Meis2 were not caused most probably by insufficient cell proliferation or changed expression of main NCC specifiers we performed whole-mount in situ hybridization to map the position of migrating NCC. At first, E9.5 embryos from Meis2-/- embryos and control littermates were stained with riboprobes for *FoxD3* and *Sox9* mRNA [46, 47]. As shown in Fig. 9A-B, both *FoxD3* and *Sox9* were expressed in Meis2-/- embryos at the levels that were comparable to control littermates, indicating again that the overall differentiation program of NCC was not affected. Interestingly, we observed an aberrant position of FoxD3-positive cranial NCC in Meis2 nulls (arrows in Fig. 9a-a’). Expression of *FoxD3* mRNA in the trigeminal ganglion confirmed its abnormality as also seen in Fig. 6b’. *Sox9* mRNA revealed that otic vesicles were consistently smaller in mutants (arrow in Fig. 9b’). Next, we carried out the same analysis in E9.5 embryos from conditional mutants AP2α-Cre/Meis2 cKO. However, the levels and distribution *FoxD3* and *Sox9* transcripts were not altered in conditional mutants (n = 5 litters, 8 mutants) (Fig. 9c-d’). Thus, the changes in the expression pattern of NCC specifying genes Sox9 and FoxD3 were found to be more profound in systemic Meis2 mutants compared to conditional ones which may explain their stronger phenotypic defects.

## Discussion

In this study, we showed that the transcription factor Meis2 is abundant in NCC and it is essential for their function. The embryonic lethal phenotypes of mutant mouse embryos of Meis2 and its paralogue Meis1 are similar in the timing of death and in strong hemorrhaging. Meis1 is expressed hematopoietic stem cells in the fetal liver, the primary organ of hematopoiesis at E13. Various assays showed defects in erythropoiesis providing explanation for anemia in Meis1 mutants [25, 26]. In contrast, we did not detect Meis2 in the fetal liver and in hematopoietic progenitors in the dorsal aorta. We therefore speculate that hemorrhaging in Meis2-/- embryos caused destructive changes primarily in the fetal liver that is highly sensitive to low oxygen. Impaired function of the liver may lead to failure in erythropoiesis and thus bleeding rather than defects in hematopoiesis in Meis2 mutants may cause lethality. Despite 83 % sequence identity of Meis1 and Meis2 and the almost identical homeodomain, they acquired different functions during evolution probably due to distinct expression patterns.

Stankunas and colleagues [15, 45] showed that various combinations of mutant alleles of Pbx1/2/3 or Meis1 exhibit cardiac anomalies in the outflow tract or in the septation of ventricles implying that cardiac NCC require transcriptional control by combinatory Meis/Pbx complexes. Along this line, zebrafish Meis2 genes influence development of heart and cranial skeleton [16, 28]. The data presented here extend our knowledge about the role of Meis/Pbx complexes controling the fate of NCC. Apart from the previously proposed function in cardiac neural crest, we suggest that a similar transcriptional control network takes place in cranial NCC including chondrogenic and neuronal lineages. Our data represent the first loss-of-function study of Meis2 in mouse and may in fact serve as a disease model for certain human developmental disorders. Intriguingly, several reports describe patients with Meis2 mutations who display disorders such as the cleft palate, septal defects in the heart or intellectual disabilities [30–33].

Pax3 is expressed in the neural plate border and plays an important role in cardiac NCC as documented by conditional knock-out studies in mouse [44]. A specific Pbx1/Meis1 complex has been reported to directly regulate Pax3 in premigratory NCC in rhombomeres, in which cardiac NCC originate, linking Pbx1-Pax3 regulatory hierarchy with the OFT defects in Pbx1-/- embryos [45]. We did not see reduced Pax3 expression in Meis2-/- at E10.5 although Pax3-positive trigeminal ganglions were shrunken in systemic mutants. Nor could we detect Pax3 in the wild-type OFT at E10.5. It is possible that Meis2 and Pax3 are co-expressed in earlier NCC in which these factors may cooperate during NCC differentiation.

Our experiments utilizing conditional mutants AP2α-Cre/Meis2 cKO confirm defects in tissues derived from cardiac and cranial NCC such as the OFT or craniofacial cartilage. However, the phenotypes in cKO appeared in some cases weaker than in systemic mutants. PTA, for instance, was never found in conditional mutants while all systemic mutants displayed defective septation of the OFT. It is important to note that we still found a substantial amount of Meis2 protein after conditional deletion in the areas that are in close vicinity of AP2α-Cre targeted regions (mapped in the ROSA26 reporter) in which we observed effective inactivation. Standby Meis2-positive cells may be involved in forming the truncus septum. Although AP2α-Cre mouse is the earliest driver for NCC [44], we cannot exclude the possibility that a minor NCC population forms before AP2α-Cre mediated recombination and thus it is not targeted. Another possibility is that neighboring non-NCC cells expressing Meis2 participate in the truncus septation. In this context it is interesting to note that systemic Pax3 KO display the PTA while Wnt1-Cre or AP2α-Cre cKOs of Pax3 show valve defects but normal separation of aorta and pulmonary arteries [44]. Moreover, the PTA defects were also seen in systemic Pbx1-3 mutants; the data from Pbx cKOs are not available [15, 45]. The fact that the truncus septation may partially originate from non-NCC population is supported by the report of Bai and colleagues [48] who observed PTA in Mef2c-Cre/Bmp4/7, a Cre driver not normally used for NCC targeting.

Altered proliferation, apoptosis or significant changes in expression of transcription factors determining NCC (Sox9, Sox10, Tfap2, Pax3, Mitf) were not detected between critical stages E10.5-E11.5 in our hands. Meis2 may influence late phases of NCC differentiation (including cell proliferation and viability) that is required for proper formation of the OFT or for differentiation of osteochondral progenitors.

We hypothesize that Meis2 absence may be compensated by Meis1 which may rescue some defects during earlier phases of NCC development. Meis2 deficiency is thus reflected in the areas and stages in which the Meis2 function is unique, e.g. during craniofacial development. Meis proteins may diversify in their expression pattern after the initial NCC specification and acquire unique function, for instance, during formation of the OFT and cartilage. In order to test this hypothesis it will be necessary to generate and analyze Meis1 and Meis2 conditional double mutants that may reveal potentially earlier role of Meis factors during NCC development.

Even though we did not see a major difference in Sox9 expression in early embryos, anomalies in the craniofacial skeleton of Wnt1-Cre/Sox9 cKO [47] and our AP2α-Cre/Meis2 cKO are similar in the mandible, tongue, the otic capsule and the hyoid bone. This suggests that Sox9 and Meis2 cooperate in a similar differentiation process during chondrogenesis. It remains to be elucidated what genes are direct targets of Meis factors. Altogether, our loss-of-function studies show that Meis2 transcription is an important player in the gene regulatory network determining differentiation of cardiac and cranial NCC.

## Methods

### Generation of Meis2 null mice

The LoxP recognition elements for the Cre recombinase were inserted in the introns 2 and 6 of the *Meis2* gene at the Gene Targeting & Transgenic Facility, University of Connecticut, USA. Transgenic mice termed Meis2 cKO were created by standard techniques using homologous recombination in mouse embryonic stem cells (129SvEvTac/C57BL/6 J F1) also at the Gene Targeting & Transgenic Facility. A neomycin selection cassette was removed using FLP-FRT recombination. Meis2 cKO were crossed to Hprt-Cre mice (strain 129S1/Sv-Hprt^tm1(cre)Mnn^/J, stock 004302, The Jackson Laboratory) with the zygotic activity of the Cre recombinase to obtain animals that were heterozygous for Meis2 (Meis2+/-) in the mixed genetic background. Primers for genotyping Meis2+/- alleles: Mrg1-lox-F (forward) GAGGGGACAGTGGGTAAACA, Mrg1-frt-R (reverse) TCAGACCCAGGAATTTGAGG, a PCR product of 256 bp. Wild-type allele: Mrg1-frt-F GCAAGGGTGCTGAGGTTAAA and Mrg1-frt-R TCAGACCCAGGAATTTGAGG, a PCR product 235 bp. (Fig. 1a). Alternatively, Mrg1-lox-F GAGGGGACAGTGGGTAAACA, Mrg1-lox-R GCGTTGCAGCTCACAAGAAT, a PCR product of 142 bp.

All procedures involving experimental animals were approved by the Institutional Committee for Animal Care and Use (permission #PP-071/2011). This work did not include human subjects.

### Immunohistochemistry

Embryos were fixed in 4 % paraformaldehyde overnight at 4 °C. 8-10 μm cryosections or 5-μm (paraffin-embedded) sections were permeabilized in 0.1 % Triton X-100 in PBS (PBT). After blocking sections were incubated overnight in a primary antibody (1 % BSA in PBT), washed with PBS and incubated with a fluorescent secondary for 1 h. Nuclei were visualized by DAPI (4,6-diamidino-2-phenylindol, 0.1 μg ml^− 1^, Roche). Primary antibodies: anti-Meis2 and anti-Meis1 (a gift from Dr. Buchberg), anti-Myl7 (1:250, Santa Cruz), smooth muscle actin (SMA) (1:1000, Sigma), Sox10, Twist1 (all Santa Cruz Biotech), 2H3 (neurofilaments), 3B5 (Tfap2a) and Pax3 (all DSHB), anti-alpha sarcomeric actin clone 5C5, anti-alpha smooth muscle actin clone 1A4 (both Sigma), anti-Myl7 (H60) (Santa Cruz Biotech), Mitf (a gift from Dr. H. Arnheiter), anti-Ter119 (BD Pharmingen). Secondary antibodies: anti-mouse (-rat, -rabbit) Alexa Fluor488 or 594 (Life Technologies). Biotinylated-anti-mouse, -anti-rabbit, -anti-rat (Vector Laboratories), Vectastain ABC Elite kit and ImmPACT DAB substrate (all Vector Laboratories). Images were acquired in Leica MZ APO stereomicroscope with DC200 camera or Olympus SZX9 with DP72 camera. Fluorescence images were acquired in Zeiss Axioskop 2 microscope with objectives Ph3 Plan-Neofluar 40x/1.3 oil or Ph1 Plan-Neofluar 10x/0.3 and confocal Leica SP5. Bright-field light images were acquired in Nikon Diaphot 300 with objectives 4x/0.1 and 10x/0.25.

#### Scale2 protocol

Dissected embryonic hearts were fixed overnight in 4 % PFA, washed in PBS and transferred into ScaleA2 reagent (4 M urea, 10 % glycerol, 0.1 % triton X-100) as described in [49]. After two hours at RT hearts were photographed.

### Alcian blue/Alizarin red staining

Embryos at E16.5-17.5 were dissected and scalded in hot water (65-70 °C, 2 min). They were dehydrated in 95 % ethanol for 48-72 h, changing solution every 12 h. After Alcian blue (Sigma) staining for 12 h, they were rinsed twice in ethanol and kept overnight. After clearing in 1 % KOH for 2 h and they were stained with Alizarin red (Sigma) for 5 h. Further clearing in 2 % KOH was carried out overnight, then in Glycerol (25 %) and 2 % KOH (75 %) for 8 h and Glycerol (50 %) and 2 % KOH (50 %) for 48 h. Tissue sections were rehydrydated and stained in 0.04 % Alcian solution for 10 min. Pictures were obtained using binocular microscope Olympus SYX9 and camera Olympus DP72.

### Whole-mount in situ hybridization

Riboprobes: Mouse Foxd3 was cloned into pGEM-T-easy vector (Promega) using primers F-GGACCGCAAGAGTTCGCGGA, R-TCCGGAGCTCCCGTGTCGTT and antisense mRNA was transcribed with T7 polymerase. Mouse Sox9 gene was cloned into pGEM-T-easy using primers F-GAGCACTCTGGGCAATCTCAG, R-CTCAGGGTCTGGTGAGCTGTG and antisense mRNA was transcribed with T7 polymerase. Whole-mount in situ hybridization was performed using standard protocols.

## Conclusions

We present a pioneering functional description of Meis2, a member of TALE-class homeodomain transcription factors, which is strongly expressed in cranial neural crest cells. We generated a conditional allele of the Meis2 gene. Using systemic and neural crest-specific inactivation of Meis2, we provide evidence that Meis2 is an important player in the regulatory network controlling cranial and cardiac neural crest cells.

## Additional file

Additional file 1: Figures S1-S4. Fetal liver in Meis2-/- at E13.5 contains less erythrocytes and loses cell viability. **Figure S2.** Meis2 is abundant in the mesenchyme of the aorta-gonadmesonephros (AGM). **Figure S3.** Meis2 is strongly expressed in migrating NCC and in mesenchymal cells at E10.5. **Figure S4.** Proliferation and cell death of migrating NCC appears normal in Meis2-/- mutants. (PDF 1406 kb)

## Abbreviations

ao: aorta
cKO: conditional knock-out
DORV: double outlet right ventricle
MO: morpholino
NCC: neural crest cells
OFT: outflow tract
pa: Pulmonary artery
PTA: persistent truncus arteriosus
EMT: epithelial-mesenchymal transtition
