# Supplementary material for: Meis2 is essential for cranial and cardiac neural crest development
Source: BMC Dev Biol. 2015 Nov 6;15:40. doi: 10.1186/s12861-015-0093-6 (PMC4636814; doi:10.1186/s12861-015-0093-6)
Supplement: Additional file 1: Figures S1-S4. — Fetal liver in Meis2-/- at E13.5 contains less erythrocytes and loses cell viability. Figure S2. Meis2 is abundant in the mesenchyme of the aorta-gonadmesonephros (AGM). Figure S3. Meis2 is strongly expressed in migrating NCC and in mesenchymal cells at E10.5. Figure S4. Proliferation and cell death of migrating NCC appears normal in Meis2-/- mutants. (PDF 1406 kb) [file 12861_2015_93_MOESM1_ESM.pdf]

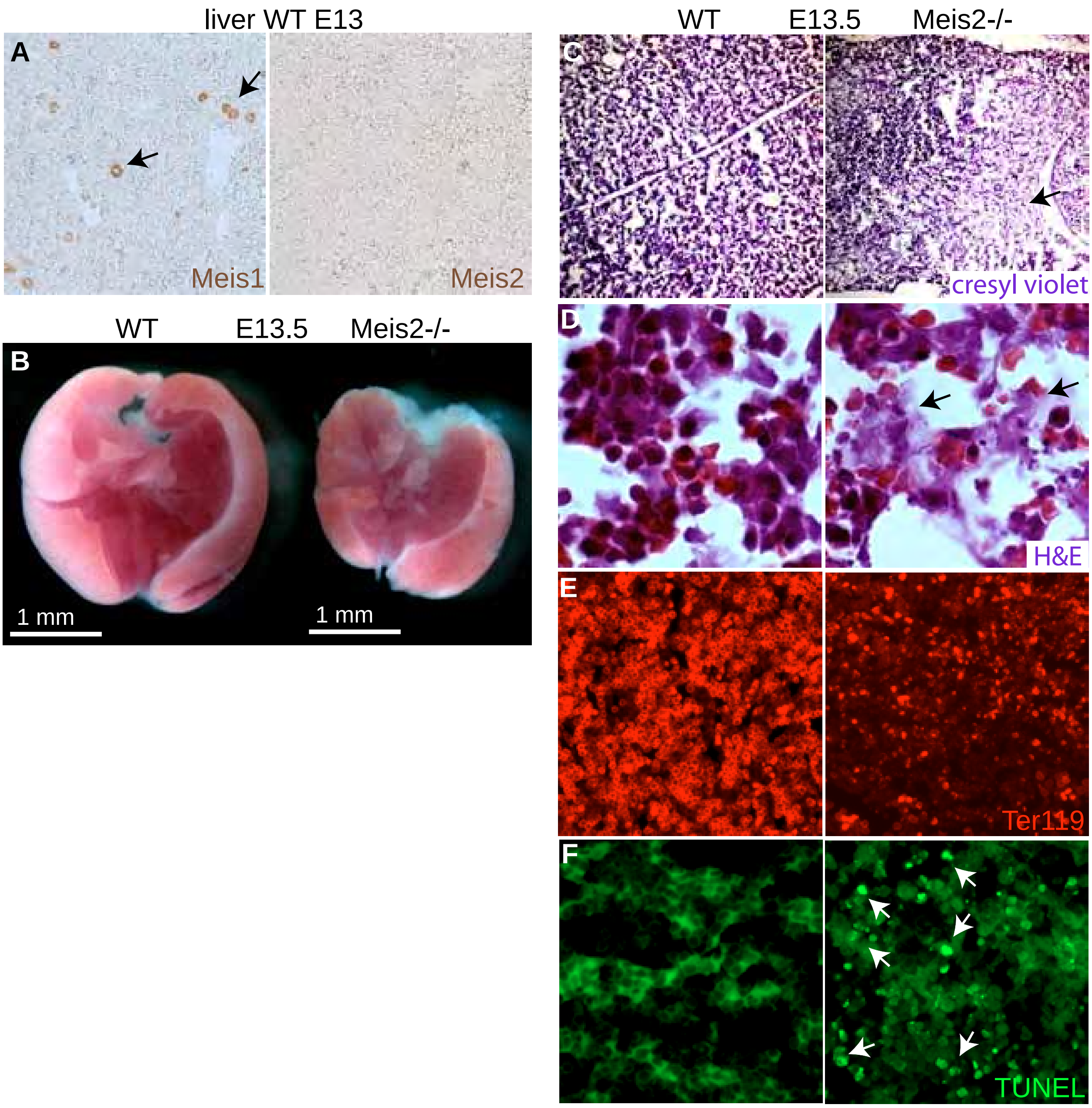

liver WT E13
A
Meis1
Meis2
WT
E13.5
Meis2-/-
B
1 mm
1 mm
C
cresyl violet
D
H&E
E
Ter119
F
TUNEL

Fig. S1

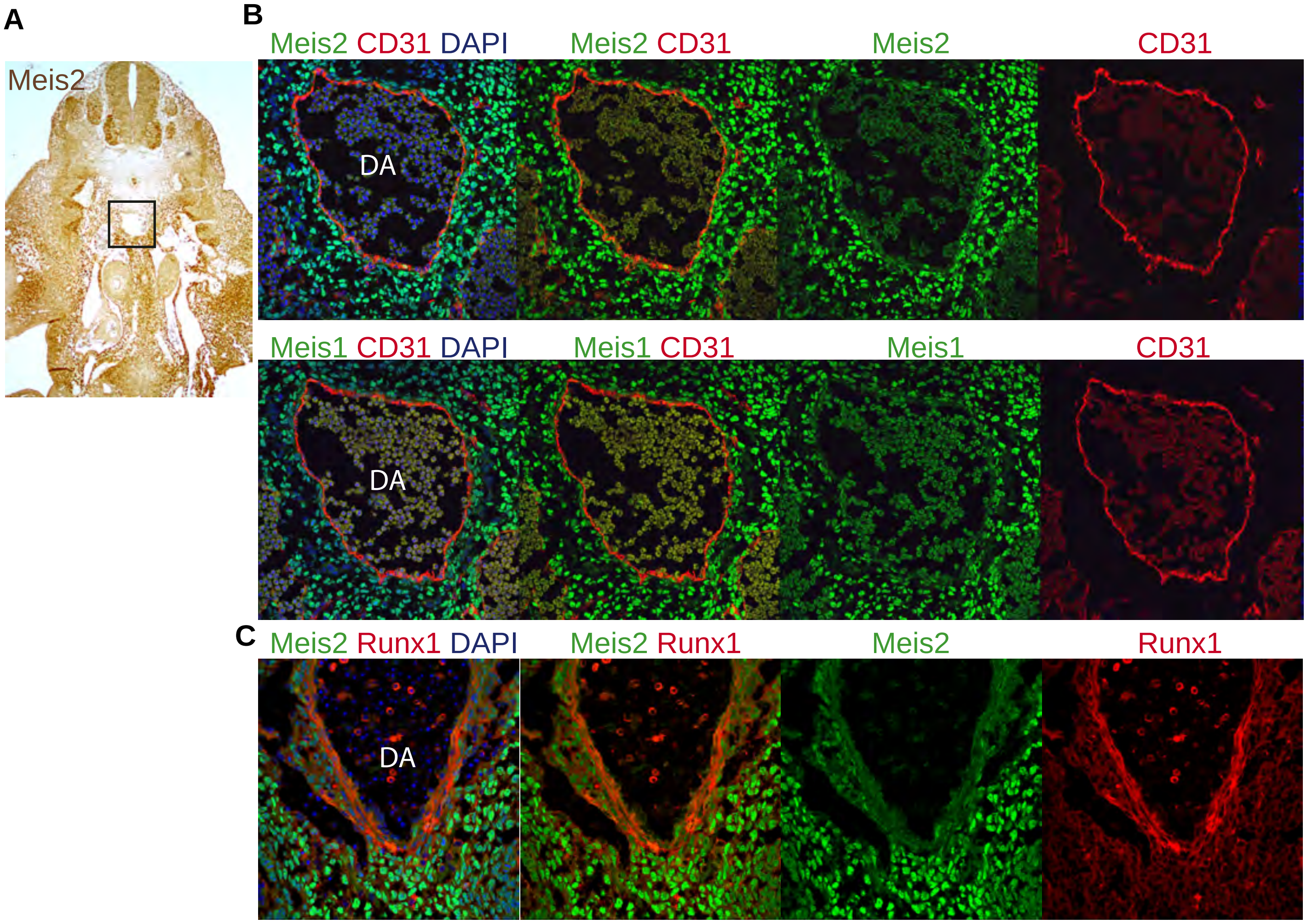
A
Meis2
B
Meis2 CD31 DAPI
Meis2 CD31
Meis2
CD31
DA
Meis1 CD31 DAPI
Meis1 CD31
Meis1
CD31
DA
C
Meis2 Runx1 DAPI
Meis2 Runx1
Meis2
Runx1
DA

Fig. S2

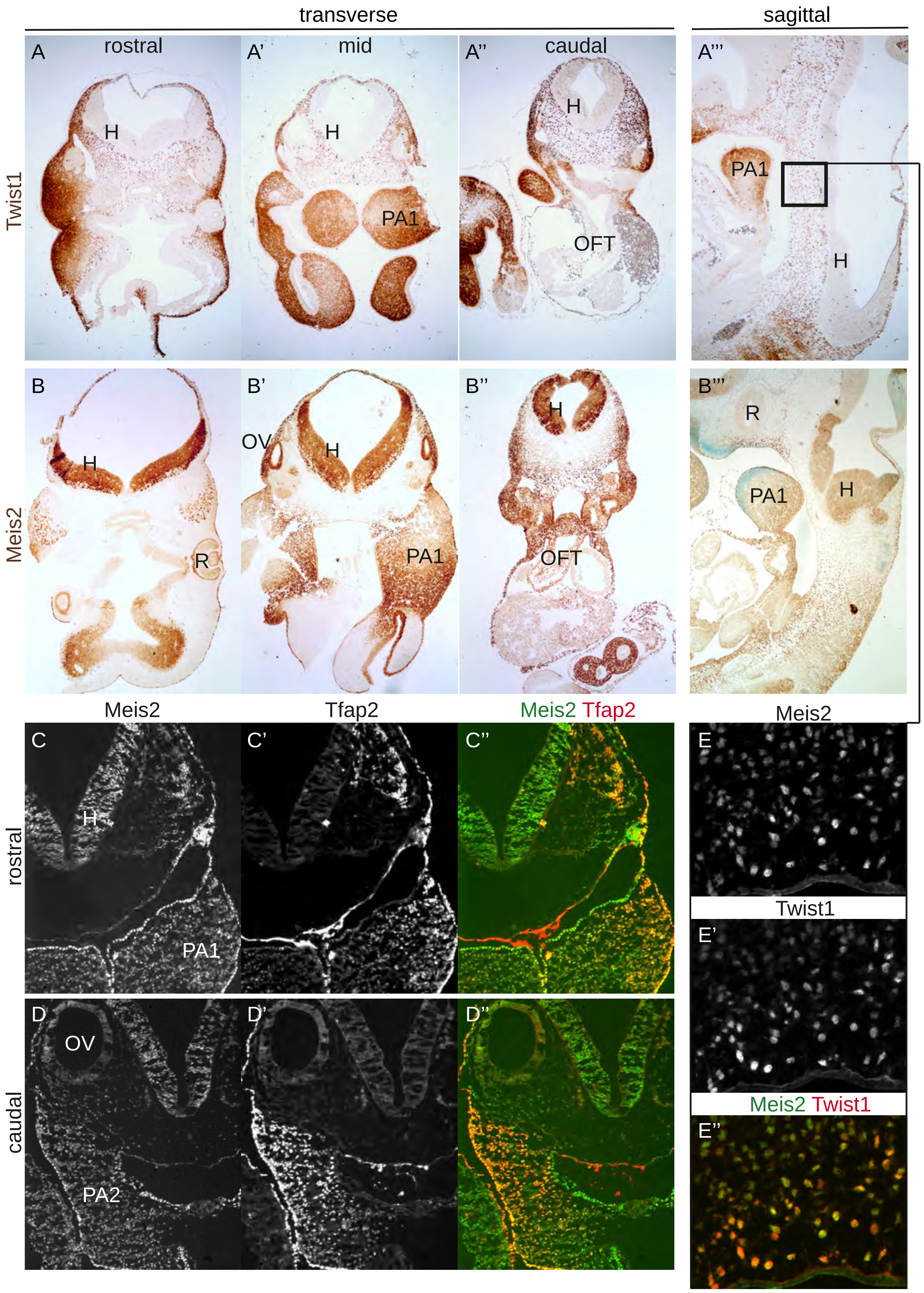
transverse
sagittal
rostral
mid
caudal
Twist1
Meis2
A
A'
A''
A'''
H
PA1
OFT
B
B'
B''
B'''
OV
R
Meis2
Tfap2
Meis2 Tfap2
C
C'
C''
rostral
caudal
D
D'
D''
PA2
E
E'
E''
Twist1
Meis2 Twist1

Fig. S3

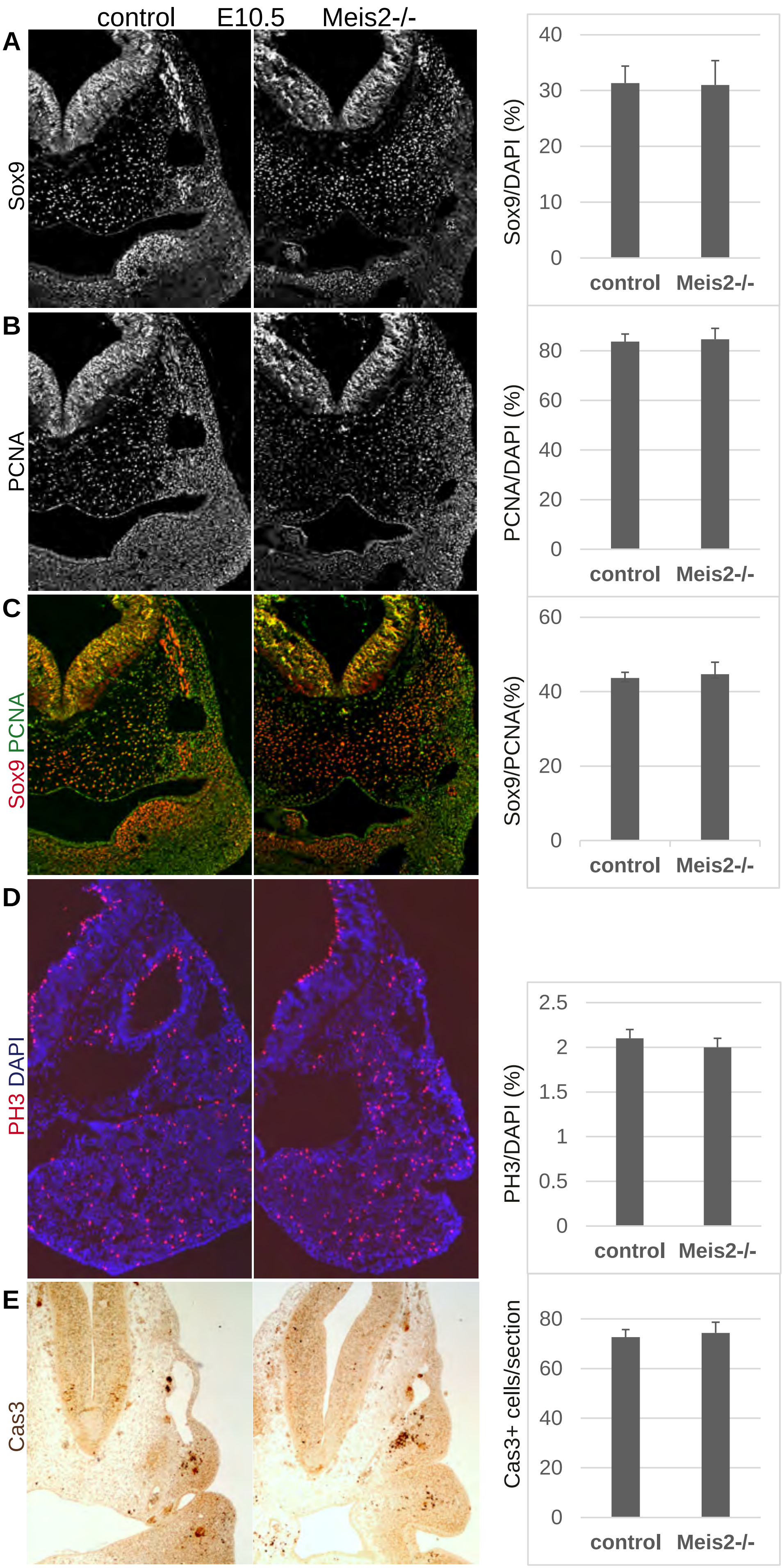
control
E10.5
Meis2-/-
A
Sox9
Sox9/DAPI (%)
40
30
20
10
0
control
Meis2-/-
B
PCNA
PCNA/DAPI (%)
80
60
40
20
0
control
Meis2-/-
C
Sox9 PCNA
Sox9/PCNA(%)
60
40
20
0
control
Meis2-/-
D
PH3 DAPI
PH3/DAPI (%)
2.5
2
1.5
1
0.5
0
control
Meis2-/-
E
Cas3
Cas3+ cells/section
80
60
40
20
0
control
Meis2-/-

Fig. S4

Figure S1. Fetal liver in Meis2-/- at E13.5 contains less erythrocytes and loses cell viability. (A) Meis2 is not expressed in the fetal liver in contrast to Meis1 that is readily detected by immunohistochemistry. **(B)** Comparison of the liver size in controls and Meis2-/-. **(C-D)** Some regions in the mutant liver show disrupted cellular network and cell viability as revealed by cresyl violet and hematoxylin/eosin staining at a higher magnification. **(E)** Erythrocyte immunofluorescence using anti-Ter119 on liver sections at E13. **(F)** Apoptosis assay on liver sections using Cell Death Detection kit (Roche).

Figure S2. Meis2 is abundant in the mesenchyme of the aorta-gonad-mesonephros (AGM). (A) An overview of Meis2 immunohistochemistry in the trunk of mouse embryo at E10.5. **(B)** Immunofluorescent double-labeling of Meis2 or Meis1 and CD31 in AGM in which both proteins are absent in the endothelial wall (CD31+) of the dorsal aorta (DA) but strongly expressed in the surrounding mesenchyme. **(C)** Immunofluorescent double-labeling of Meis2 and Runx1 in the dorsal aorta at the AGM region showing no expression of Meis2 in circulating hemapoietic stem cells.

Figure S3. Meis2 is strongly expressed in migrating NCC and in mesenchymal cells at E10.5. (A-A'') Rostral to caudal transverse sections at a low-power magnification show the presence of Twist1 in mesenchymal cells around the otic vesicle, in PA1-PA2 and in the aortic sac. (A''') Sagittal view on E10.5 embryo stained for Twist1. **(B-B'')** Rostral to caudal transverse sections at a low-power magnification show Meis2 expression at E10.5 in mesenchymal cells, neuroectoderm, otic vesicle, PA1-PA2 and heart, (B''') sagittal view on Meis2 pattern. **(C-D)**

Immunofluorescent double-labeling of Meis2 and Tfap2 in a rostral region with PA1 (C-C') and in a caudal region with otic vesicle (D-D'') demonstrating a high number of NCC co-expressing both proteins. **(E-E'')** Immunofluorescent double-labeling of Meis2 and Twist1 in the region depicted by rectangle in (A'''). F, forebrain; H, hindbrain; OFT, outflow tract; OV, otic vesicle; PA1-2, pharyngeal arches; R, retina.

Figure S4. Proliferation and cell death of migrating NCC appears normal in Meis2-/- mutants. (A-C) Sox9 and PCNA alone and double labeling (C) of transverse sections in the region of the otic vesicle in Meis2-/- E10.5 embryos. Corresponding quantifications (as the percentage relative to all DAPI cells on a section) are shown to the right. **(D)** PH3 immunofluorescence and its counting with no significant difference in proliferating cells in the mutants. **(E)** Quantification of apoptosis assayed as the number of Cas3-positive cells after immunostaining of embryonic sections at E10.5 (brown). Bars represent the average from three experiment with standard deviations.
